# Critical role of p38 MAPK for regeneration of the sciatic nerve following crush injury *in vivo*

**DOI:** 10.1186/1742-2094-10-1

**Published:** 2013-01-03

**Authors:** Naoki Kato, Masahito Matsumoto, Masakazu Kogawa, Gerald J Atkins, David M Findlay, Takahiko Fujikawa, Hiromi Oda, Masato Ogata

**Affiliations:** 1 Department of Orthopaedic Surgery, Saitama Medical University, Saitama, Japan; 2 Department of Orthopaedic Surgery, Saitama Medical Center, Saitama Medical University, 1981 Kamoda, Kawagoe City, Saitama, 350-8550, Japan; 3 Department of Molecular Biology, Saitama Medical University, Saitama, Japan; 4 Division of Functional Genomics & Systems Medicine, Research Center of Genomic Medicine, Saitama Medical University, Saitama, Japan; 5 Discipline of Orthopaedics and Trauma, The University of Adelaide, Adelaide, Australia; 6 Department of Biochemistry, Mie University School of Medicine, Mie, Japan

**Keywords:** P38 MAPK, Nerve regeneration, Crush injury, Inflammatory cytokines, TNF-α, IL-1β, Mutant mice, *In vivo*

## Abstract

**Background:**

The physiological function of p38α, which is an isoform of p38 MAPK, has been investigated previously in several studies using pharmacological inhibitors. However, the results regarding whether p38α promotes or inhibits nerve regeneration *in vivo* have been controversial.

**Methods:**

We generated novel p38α mutant mice (sem mice) with a point mutation in the region encoding the p38α substrate-docking-site, which serves as a limited loss-of-function model of p38α. In the present study, we utilized sem mice and wild-type littermates (wt mice) to investigate the physiological role of p38α in nerve regeneration following crush injuries.

**Results:**

At four weeks after crush injury, the average axon diameter and the average axon area in sem mice were significantly smaller than those in wt mice. The average myelin sheath thickness in sem mice was reduced compared to wt mice, but no significant difference was observed in the G-ratio between the two groups. The sciatic functional index value demonstrated that functional nerve recovery in sem mice following crush injury was delayed, which is consistent with the histological findings. To investigate the underlying mechanisms of these findings, we examined inflammatory responses of the sciatic nerve by immunohistochemistry and western blotting. At an early phase following crush injury, sem mice showed remarkably lower expression of inflammatory cytokines, such as TNF-α and IL-1β, than wt mice. The expression of Caspase-3 and Tenascin-C were also lower in sem mice. Conversely, at a late phase of the response, sem mice showed considerably higher expression of TNF-α and of IL-1β with lower expression of S-100 than wt mice.

**Conclusions:**

This is the first study of the physiological role of p38 MAPK in nerve regeneration that does not rely on the use of pharmacological inhibitors. Our results indicate that p38α insufficiency may cause an inflammatory disorder, resulting in a delay of histological and functional nerve recovery following crush injury. We conclude that p38 MAPK has an important physiological role in nerve regeneration and may be important for controlling both initiation of inflammation and recovery from nerve injury.

## Background

It is well known that the peripheral nervous system is remarkable in its ability to regenerate after injury. In the transected or crushed peripheral nerve, the distal segment undergoes Wallerian degeneration, which is essential for the subsequent processes of nerve regeneration [1]. Therefore, understanding the molecular mechanisms between nerve degeneration and regeneration holds the key to further advances in the clinical management of such injuries. In the event of nerve regeneration, cytokines have been considered to be critical components, since these are important mediators of communication between various types of cells. Furthermore, it has been reported that expression of cytokines, including pro- and anti-inflammatory cytokines, are controlled in a highly ordered fashion during Wallerian degeneration and subsequent regeneration in the peripheral nerve system [2,3]. Of these pro-inflammatory cytokines, TNF-α is upregulated early and transiently at the site of nerve injury and is considered to play a crucial role in the process of Wallerian degeneration as an initiator of local inflammatory responses [2,4]. However, the complex signaling pathways controlling these events remain unknown. Further understanding of the cell and molecular program controlling the expression of inflammatory cytokines following nerve injuries is important in order to devise ways to promote nerve regeneration.

Mitogen-activated protein kinase (MAPK) cascades are highly conserved signal transduction pathways coupling different extracellular signals to a variety of intracellular responses [5]. The signaling pathway is similar for all the members of the MAPK family and is typically composed of a highly conserved MAPK module comprising three kinases, namely MAPK kinase kinase (MKKK), MAPK kinase (MKK) and MAPK. The p38 MAPK is a member of this MAPK family of serine-threonine kinases, which is specifically activated by phosphorylation under conditions of hypoxia, stress, and exposure to inflammatory cytokines [6]. Activated p38 MAPK subsequently phosphorylates its substrates, including protein kinases such as MAPKAP kinase-2 and several transcription factors including MEF2, CHOP, and ATF-2, leading to the induction of cell proliferation and/or apoptosis [7]. In this pathway, dephosphorylation of either Thr180 or Tyr182 is sufficient to inactivate p38 MAPK, and this can be mediated by tyrosine-specific MAPK phosphatases. The MAPK cascade can also induce phosphatase gene transcription, providing a negative feedback for MAPK activation.

It has been reported that p38 MAPK has four isoforms, p38α, p38β, p38γ, and p38δ, which share 60% to 70% amino acid sequence identity. Although these isoforms have overlapping substrate specificity, some substrates appear to be preferentially phosphorylated by one or more isoforms [8]. Of these isoforms, p38α is expressed ubiquitously and has been proposed to regulate many cellular processes. In the nervous system, it has been reported that p38α plays critical roles in the differentiation and/or survival of neurons, regulating neural plasticity and inflammatory responses. Considering that inflammatory responses induced by nerve injury are indispensable for subsequent nerve regeneration, further investigation of the p38α signaling pathway may be a key to understanding the mechanisms regulating nerve regeneration.

There are a large number of reports regarding p38α function in cultured cells. However, very little is known about the physiological role of this MAPK *in vivo*, since p38α is essential for mammalian embryonic development and loss of p38α causes embryonic death [9,10]. *In vivo* studies to investigate p38 MAPK function have usually been undertaken using pharmacological inhibitors, such as SB 203580, which targets both p38α and p38β [11]. However, the physiological function of p38 MAPK *in vivo* remains controversial. Temporin *et al*. reported that SB 203580 reduced axonal length in a neurite outgrowth assay. In that study, the activation of RhoA appeared to be responsible for this effect, since the addition of SB 203580 increased RhoA activity to 2.1-fold compared with control group [12]. On the other hand, Myers *et al*. found that SD-169, a novel oral inhibitor of p38 MAPK, increased the rate of nerve fiber regeneration following peripheral nerve crush injury [13]. The reasons for these conflicting results are not entirely clear, however, we surmise that the specificity of these chemical inhibitors, critically depending on their concentration, might be a contributing factor. Godl *et al*. studied the selectivity of SB 203580, widely used as a p38 MAPK inhibitor, using a proteomics approach and reported that RICK (Rip-like interacting caspase-like apoptosis-regulatory protein kinase/RIP2/CARDIAK) was even more potently inhibited by SB 203580 than p38α. Since RIP2/RICK/CARDIAK is a member of the receptor interacting protein kinase (RIP) family and promotes NF-κB activation as well as activation of the MAPKs JNK, ERK1/2 and p38 MAPK, these authors commented that incorrect conclusions might have been drawn from numerous experiments, in which SB 203580 was used as a ‘specific’ p38 inhibitor [14]. Thus, to understand the precise physiological functions of p38α *in vivo*, more specific methods than pharmacological inhibition are required.

It has been reported that docking interactions of MAPKs via docking domains are important in regulating both the activation and inactivation of these kinases, and mutation of this docking domain in p38α/p38β disrupted the p38 docking interaction, resulting in dysfunction of p38α and p38β [15]. In this domain, two aspartate acids are essential for docking and the so-called *sevenmaker* mutant, in which one of these two aspartate acids is changed, showed markedly decreased kinase activity on MAPK phosphatase-1 *in vitro*[16,17].

Recently, we generated novel p38α mutant mice with a point mutation in the p38α substrate-docking-site. Using the Cre/lox-conditional mutation system, we converted aspartate acid at position 316 in exon 11 of p38α to asparagine. As a result, these mutant mice, termed sem (*sevenmaker* type Mapk14) mice, possess a knock-in mutation in p38 MAPK (D316N) [18]. In the present study, we utilized these sem mice and wild-type littermates (wt mice) to investigate the physiological role of p38α during nerve regeneration following crush injury.

## Materials and methods

### Animals

Sem C57BL/6N mice were bred with wt C57BL/6N mice. Approximately half of the resulting offspring carried the mutant p38α gene, as confirmed by polymerase chain reaction (PCR) genotyping with primers specific for the mutant p38α gene, 5^′^-TAG ATA CAG AGC CAT CAG ACC ACC A-3^′^ (sense primer) and 5^′^-TGA ATG GTG TAG CAT AGG CTG GA-3^′^ (antisense primer), applied to total cellular DNA isolated from tail snip tissue. Adult, male, sem mice with heterozygous mutant p38α gene (p38+/−) (12 to 16 weeks old, weighing 13 to 22 g) and wt littermates (p38+/+) (12 to 16 weeks old, weighing 17 to 27 g) were housed on a 12-hour light/dark cycle with *ad libitum* access to food and water. Body weights of both sem mice and wt mice were measured weekly. Both genotypes continued to steadily increase their weight but wt mice were measurably larger than that of sem mice throughout the experimental periods. This study was carried out in accordance with the recommendations in the Guide for the Care and Use of Laboratory Animals published by the National Institutes of Health, and the protocol was approved by the Committee on the Ethics of Animal Experiments of Saitama Medical University (approved number 673).

### Nerve crush injury model

All surgical procedures were carried out under sodium pentobarbital anesthesia (30 to 50 mg/kg, injected intraperitoneally). The left sciatic nerve was exposed through a gluteal muscle-splitting approach. A crush injury was then applied to the nerve at 5 mm distal to the sciatic notch using a brain aneurysm clip (Sugita clip; Mizuho Ikakogyo, Tokyo, Japan). The clip was left in place for three minutes with a holding force of approximately 250 g.

Twenty mice were divided into two equal groups: p38α mutant mice (sem mice; n = 10) and wild-type littermate mice (wt mice; n = 10). These mice were assessed by immunohistochemistry (see below) for the expression of TNF-α, IL-1β, Caspase-3 and Tenascin-C at three days after crush injury. Functional evaluation of nerve recovery and histological assessment including the expression of TNF-α, IL-1β and of S-100 at four weeks after crush injury were investigated through the use of another cohort of mice (n = 10 per group).

### Histological studies

At four weeks after crush injury, specimens of the crushed sciatic nerve were taken at 5 mm distal to the injury site and fixed with PBS containing 1.85% paraformaldehyde (PFA, pH 7.4) and 0.25% glutaraldehyde, as described previously [19,20]. As controls, tissue specimens were also taken from the contralateral uninjured sciatic nerve. All specimens were post-fixed with 2% osmium tetroxide, dehydrated in serially increasing concentrations of alcohol and ether, and then embedded in Epon resin (Quetol 512; Nisshin EM, Tokyo, Japan). Each section (1 μm in thickness) was stained with toluidine blue and examined under a light microscope. For the morphometric study of the sciatic nerve, three consecutive sections of one nerve were chosen, and a set of three non-overlapping fields was evaluated using random sampling from each section. The evaluated area covered approximately two-thirds of the total area of one nerve section. These were analyzed using ImageJ software (ver. 1.38, NIH, http://rsbweb.nih.gov/ij/).

Morphometric measurements of the sciatic nerve included: 1) axon (fiber without myelin sheath) diameter distribution; 2) average axon diameter (μm); 3) average axon area (μm^2^); 4) average axon number (N); 5) average axon density (N/mm^2^); 6) average G-ratio (quotient axon diameter / fiber diameter, a measure of the degree of myelination); and 7) average myelin sheath thickness (μm).

### Functional studies

All animals underwent walking track analysis, as described previously [21]. Paw prints were recorded by moistening the hind paws of each animal with blue ink and having them walk unassisted along a 6 × 44 cm corridor underlain with white construction paper. All tracks were analyzed in a blinded fashion before crush injury (0 week), and 1, 2, 3, 4 weeks following crush injury. Prints for measurement were chosen for clarity and consistency at a point when the mouse was walking at a moderate pace. If necessary, the animals were walked multiple times in order to obtain measurable prints. The tracks were evaluated for two different parameters: toe spread (TS), being the distance between the first and fifth toes, and print length (PL), the distance between the third toe and the hind pad. Measurements of all the parameters were made for the right (control) and the left (experimental) paw prints, and the sciatic functional index (SFI) was calculated according to the formula: SFI = 118.9 (ETS - NTS) / NTS - 51.2 (EPL - NPL) NPL - 7.5, where ETS is experimental TS, NTS is control TS, EPL is experimental PL and NPL is control PL.

The SFI value characterizes crucial aspects of locomotion activities, involving recovery of hind limb sensory and motor function. It varies from 0 to −100, with 0 corresponding to normal function and −100 corresponding to complete dysfunction.

### Immunohistochemical studies

Immunohistochemical examination of the nerve was performed at both three days and four weeks after crush injury. Specimens were taken from the sciatic nerve at 5 mm distal to the crush lesion, and fixed for three hours followed by cryoprotection in 30% sucrose for six hours. They were subsequently embedded in optimal cutting temperature (OCT) compound and were frozen at −80°C. Serial sagittal sections of 20 μm were cut on a cryostat and mounted on silane-coated slides. After washing with PBS, they were treated with 0.3% H_2_O_2_ in 90% methanol for 30 minutes to inactivate endogenous peroxidase. The sections were rinsed with PBS for 30 minutes and treated with 10% normal goat blocking serum in PBS for one hour at room temperature to block non-specific protein binding. Sections were then incubated overnight at room temperature in a humidified chamber with antibody. Polyclonal rabbit anti TNF-α and monoclonal mouse anti S-100 antibodies were purchased from Abcam (Cambridge, MA, USA). Polyclonal rabbit anti IL-1β antibody was purchased from Santa Cruz Biotech (Santa Cruz, CA, USA). Polyclonal rabbit anti Caspase-3 antibody and rabbit anti Tenascin-C antibody were purchased from Lifespan Bioscience (Seattle, WA, USA). The secondary antibody and detection systems were Vectastain ABC kit and DAB peroxidase substrate kit (Vector Lab., Burlingame, CA, USA). Furthermore, the sections for S-100 immunohistochemistry were counterstained with hematoxylin to evaluate cellularity.

For the immunohistochemical study of the sciatic nerve, specimens were analyzed at 400x magnification and a density threshold was set for each section to identify the immunopositive area using ImageJ software (ver. 1.38, NIH). The ratio of the immunopositive area to the total area of the field was calculated as the immunopositive rate (%). For the evaluation of cellularity, the number of cells per microscopic field was counted and expressed as the cell density (N/mm^2^). These analyses were performed using three non-overlapping microscopic fields, which were randomly selected from each section. The evaluated area covered approximately two-thirds of the total area of one nerve section. All histological and immunohistochemical evaluations were performed by an investigator who was blinded to the genotype and injury status of the mice.

### Western blot analysis

Western Blotting of sciatic nerve protein was performed at four weeks after crush injury. Specimens were taken from the sciatic nerve at 5 mm distal to the crush lesion and from the contralateral uninjured sciatic nerve. These specimens were homogenized in lysis buffer (20 mM Hepes, pH 7.4, 2 mM EGTA, 50 mM β-glycerophosphate, 0.1% Triton X-100, 10% glycerol, 1 mM dithiothreitol, 1 μg/ml leupeptin, 5 μg/ml aprotinin, 1 mM phenylmethylsulfonyl fluoride, 1 mM sodium orthovanadate). After centrifugation at 10,000 × *g* for 15 minutes at 4°C, the supernatants were electrophoresed on a 10% SDS-polyacrylamide gel and blotted onto polyvinylidene difluoride membranes. Immunoblot detection was performed with the corresponding polyclonal rabbit anti TNF-α antibody (Abcam) and polyclonal rabbit anti IL-1β antibody (Santa Cruz Biotech) using a ECL detection kit (Amersham Pharmacia Biotech, Buckinghamshire, UK). The membranes were then stripped and reprobed with polyclonal rabbit anti-β-actin antibody (Santa Cruz Biotech), which was used as control for protein loading. Band intensities at each time point (n = 3 samples per group) were analyzed and quantified as relative density against control.

### Statistical analysis

All values of morphometric parameters from histological studies, functional studies, immunohistochemical studies and western blot analysis were expressed as means ± S.D. All statistical analyses were assessed using Student’s *t* test. A value for *P* < 0.05 was considered to be statistically significant.

## Results

### Histological studies

**Figure 1:**
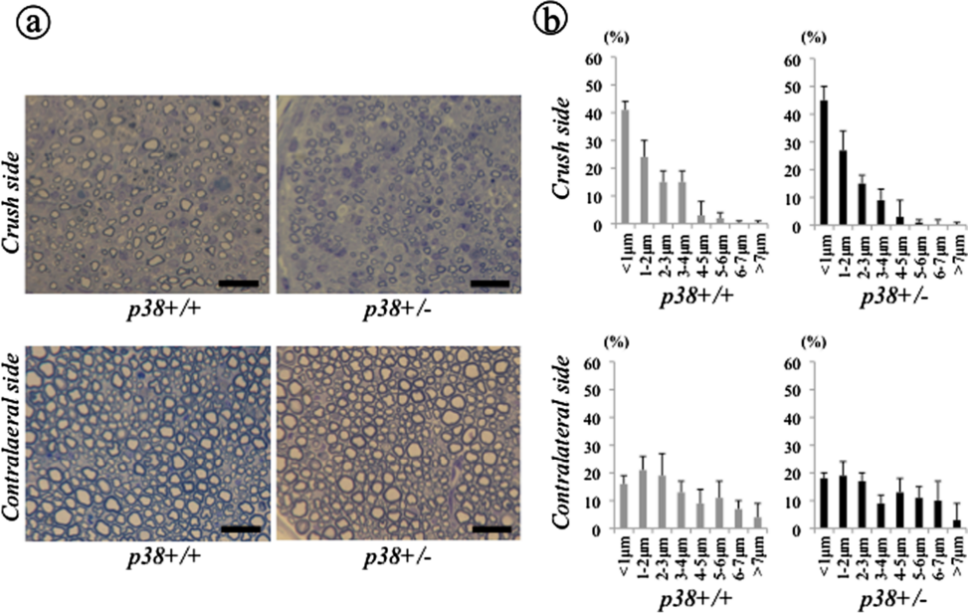
**Histological assessment of nerve regeneration at four weeks after crush injury.** At four weeks after crush injury, specimens of the crushed sciatic nerve were taken at 5 mm distal to the injury site from both sem (p38+/−) and wt (p38+/+) mice. As controls, tissue specimens were also taken from the contralateral uninjured sciatic nerve. **(a)** A 1 μm slice section was stained with toluidine blue and examined under a light microscope. Scale bar indicates 50 μm. **(b)** Axon diameter distribution of the sciatic nerve was evaluated using a set of three non-overlapping microscopic fields.

Results from previous studies using pharmacological inhibitors to investigate whether p38α promotes or inhibits nerve regeneration *in vivo* have been controversial. Therefore, we first evaluated nerve regeneration after crush injury. Baseline differences in nerve development due to the p38α mutation were determined by taking specimens from the contralateral uninjured sciatic nerve of both sem and wt mice. Both groups showed a dense axon population with many large-diameter axons with a thick myelin sheath (Figure 1a). Morphometric parameters of the contralateral sciatic nerve were not different between the two groups (Table 1). Four weeks following crush injury, many small-diameter axons with a thin myelin sheath were observed in both groups, although large-diameter axons were coexisting in wt mice. Axon diameter distribution showed increased proportion of small-diameter axons (<1 μm), which were similar between the two groups (Figure 1b), Morphometric parameters of the crushed sciatic nerve showed that the average axon diameter and area in sem mice were significantly smaller than that in wt mice (*P* < 0.05, *P* < 0.01, respectively). The average myelin sheath thickness in sem mice was also significantly reduced after crush injury compared to wt mice (*P* < 0.05), however, no significant differences were observed in the axon number, density and G-ratio between the two groups (Table 1). These findings suggest that inhibition of p38α influences nerve regeneration rather than neural development. Therefore, we consider that these sem mice are a suitable model, in which to investigate the physiological role of p38 MAPK in nerve regeneration.

**Table 1:** Morphometric parameters of the nerve at four weeks after crush injury

	**Crush side**			**Contralateral side**		
	***p38*+/+**	***p38*+/−**	***P***	***p38*+/+**	***p38*+/−**	***P***
Axon diameter (μm^2^)	1.52±0.06	1.37±0.04^*^	<0.05	3.87±1.37	3.75±1.64	N.S.
Axon area (μm^2^)	5.24±0.36	3.15±0.15^**^	<0.01	10.21±2.34	9.93±2.94	N.S.
Axon number (N)	2850±550	2700±650	N.S.	5800±300	5650±250	N.S.
Axon density (N/mm^2^)	11500±4100	10150±4650	N.S.	22450±3050	21050±2800	N.S.
G-ratio	0.86±0.05	0.87±0.04	N.S.	0.74±0.03	0.75±0.05	N.S.
Myelin sheath thickness (μm)	0.18±0.06	0.15±0.13^*^	<0.05	0.98±0.29	0.96±0.33	N.S.

N.S., not significant.

### Functional studies

**Figure 2:**
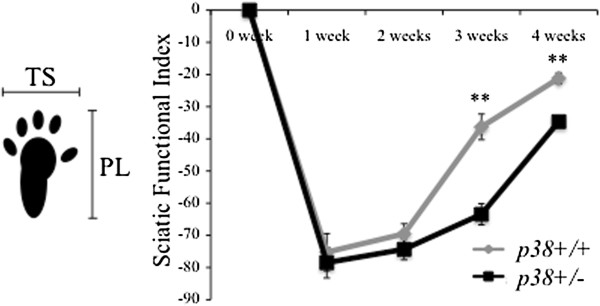
**Functional nerve regeneration after crush injury.** The sciatic functional index (SFI) was calculated before crush injury (0 week), and 1, 2, 3, 4 weeks following crush injury using two different parameters: toe spread (TS) and print length (PL). Asterisks indicate significant time point-specific differences (**P <0.01).

To evaluate total nerve regeneration *in vivo*, we applied SFI, which is an index of functional nerve recovery. Until two weeks after crush injury, SFI values showed no significant differences between wt and sem mice. However, the SFI value in sem mice was significantly lower than that in wt mice at three and four weeks after crush (*P* < 0.01) (Figure 2). This result demonstrates that functional nerve recovery of sem mice following crush injury was delayed, which is consistent with the histological findings.

### Immunohistochemical studies

Activation of p38 MAPK, and proinflammatory cytokines such as TNF-α and IL-1β, is considered to be regulated by interactions between these cytokines. It has been reported that systemic administration of p38 inhibitors results in reduced synthesis of TNF-α and IL-1β [22], and that p38 MAPK is activated by TNF-α [6], leading to induction of cell proliferation and/or apoptosis [7]. TNF-α is also known to activate p38 MAPK in Schwann cells, by upregulating the expression of IL-1β and increasing nerve growth factor (NGF) secretion [13,23]. The relationship between TNF-α and p38 MAPK is one of positive feedback in that TNF-α phosphorylates p38 MAPK, resulting in upregulation of TNF-α biosynthesis in the same cell type [13]. To investigate the effect of p38 MAPK on cytokine expression after crush injury, we determined the expression of TNF-α and IL-1β in the sciatic nerve using immunohistochemistry.

**Figure 3:**
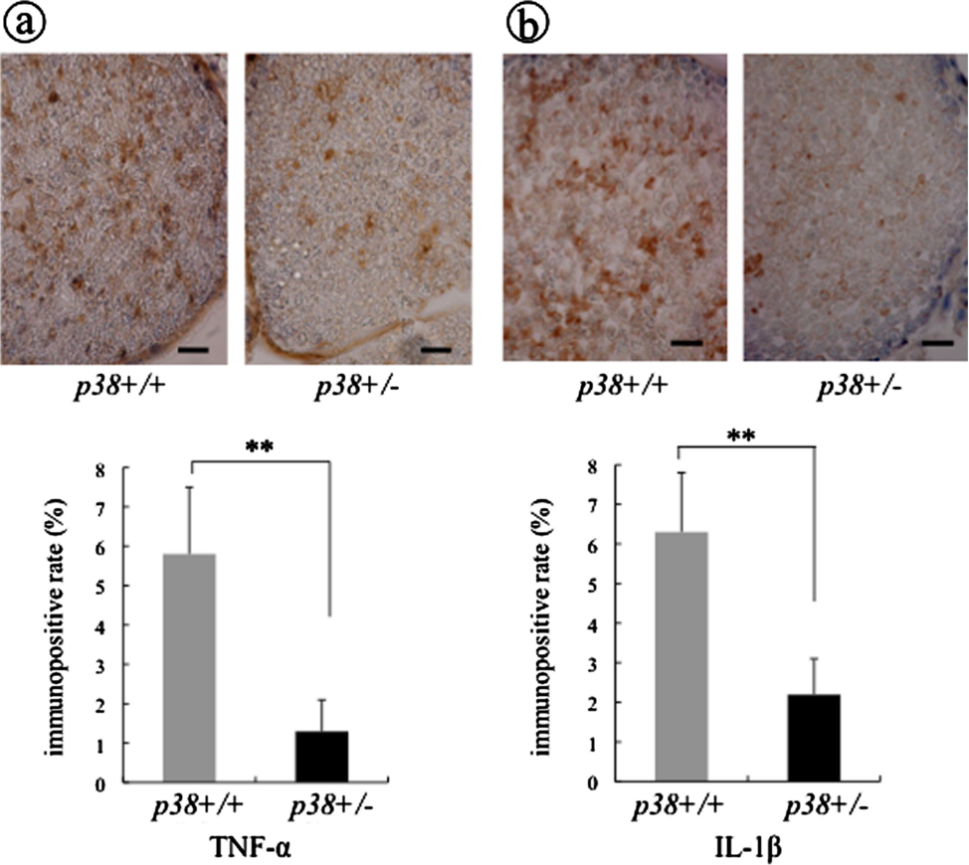
**The expression of TNF-α and IL-1β at three days after crush injury.** At three days after crush injury, specimens of the crushed sciatic nerve were taken at 5 mm distal to the injury site from both sem (p38+/−) and wt (p38+/+) mice. Each section was processed for immunostaining using anti TNF-α antibody, and anti-IL-1β antibody. Scale bar indicates 50 μm. The ratio of the immunopositive area of **(a)** TNF-α and **(b)** IL-1β to the total area of the field was calculated as the immunopositive rate, respectively. Asterisks indicate significant difference between genotypes (**P <0.01).

In the early phase (three days after injury), strong TNF-α immunoreactive (−ir) lesions spread over the entire area of the nerve bundle in wt mice, however sem mice showed fewer stained TNF-α-ir lesions. The TNF-α positive rate in sem mice was 1.3 ± 0.8%, which is significantly lower (*P* < 0.01) than that of wt mice (5.8 ± 1.7%) (Figure 3a). The number of intense IL-1β-ir lesions was also lower in the sciatic nerves taken from sem mice compared with that from wt mice. The IL-1β positive rate of sem and wt mice was 2.2 ± 0.9% and 6.3 ± 1.5%, respectively, which also represents a significant difference (*P* < 0.01) (Figure 3b). In the contralateral uninjured sciatic nerve, TNF-α-ir lesions and IL-1β-ir lesions were completely absent endoneurially in both sem and wt mice (data not shown).

**Figure 4:**
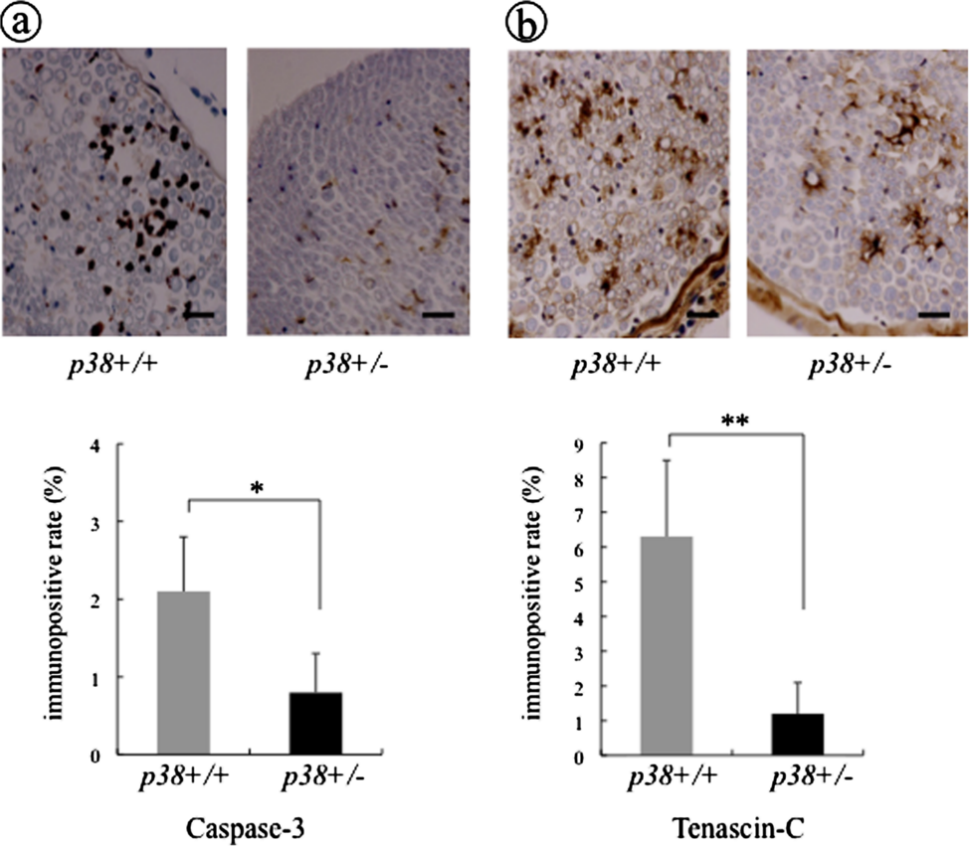
**The expression of Caspase-3 and Tenascin-C at 3 days after crush injury.** At three days after crush injury, specimens from both sem (p38+/−) and wt (p38+/+) mice were processed for immunostaining using anti Caspase-3 antibody, and anti Tenascin-C antibody. Scale bar indicates 50 μm. The ratio of the immunopositive area of **(a)** Caspase-3 and **(b)** Tenascin-C to the total area of the field was calculated as the immunopositive rate, respectively. Asterisks indicate significant difference between genotypes (*P <0.05, **P <0.01).

We further investigated the process of Wallerian degeneration, which is known to be an apoptotic phenomenon of peripheral nerves following traumatic injury and is essential for subsequent nerve regeneration. To investigate this process in the early phase after injury, we observed the expression of Caspase-3, which was reported to be expressed in Schwann cells after nerve injury [24] and to be activated in the effector phase of the apoptotic process leading to cell death within a few hours [25]. It is considered that the expression of Caspase-3 represents a reliable tool for the identification of Wallerian degeneration. At three days after nerve injury, intense Caspase-3-ir lesions were found in the entire area of the endoneurium in the sciatic nerves in wt mice, but were rarely found in sem mice. When quantified, the Caspase-3 positive rates in sem and wt mice were 0.8 ± 0.5% and 2.1 ± 0.7%, respectively, representing a significantly lower positive rate in sem mice (*P* < 0.05) (Figure 4a). By contrast, the contralateral nerve specimens taken from both sem mice and wt mice showed no expression of Caspase-3 (data not shown).

Next, we investigated the expression of Tenascin-C, an oligomeric glycoprotein of the extracellular matrix, which is diffusely expressed during neurogenesis in the peripheral nervous system [26]. It is also reported that the expression of Tenascin-C occurs within two days after removal of epiperineurium as part of the regenerative response [27] and its upregulation is an important prerequisite for axonal regrowth after peripheral nerve injury [28]. At three days after nerve injury, intense Tenascin-C-ir lesions were found in the entire area of the endoneurium in the sciatic nerves in wt mice but fewer lesions were observed in sem mice. The Tenascin-C positive rates in sem and wt mice were 1.2 ± 0.9% and 6.3 ± 2.2%, respectively, which was significantly different (*P* < 0.01) (Figure 4b). In the contralateral nerve specimens, the expression of Tenascin-C was observed only in the perineurium (data not shown). Together, these results indicate that p38 MAPK might cause TNF-α and IL-1β upregulation in the crush injured nerve lesion and play a positive role for axonal regrowth following Wallerian degeneration at early phase after injury.

**Figure 5:**
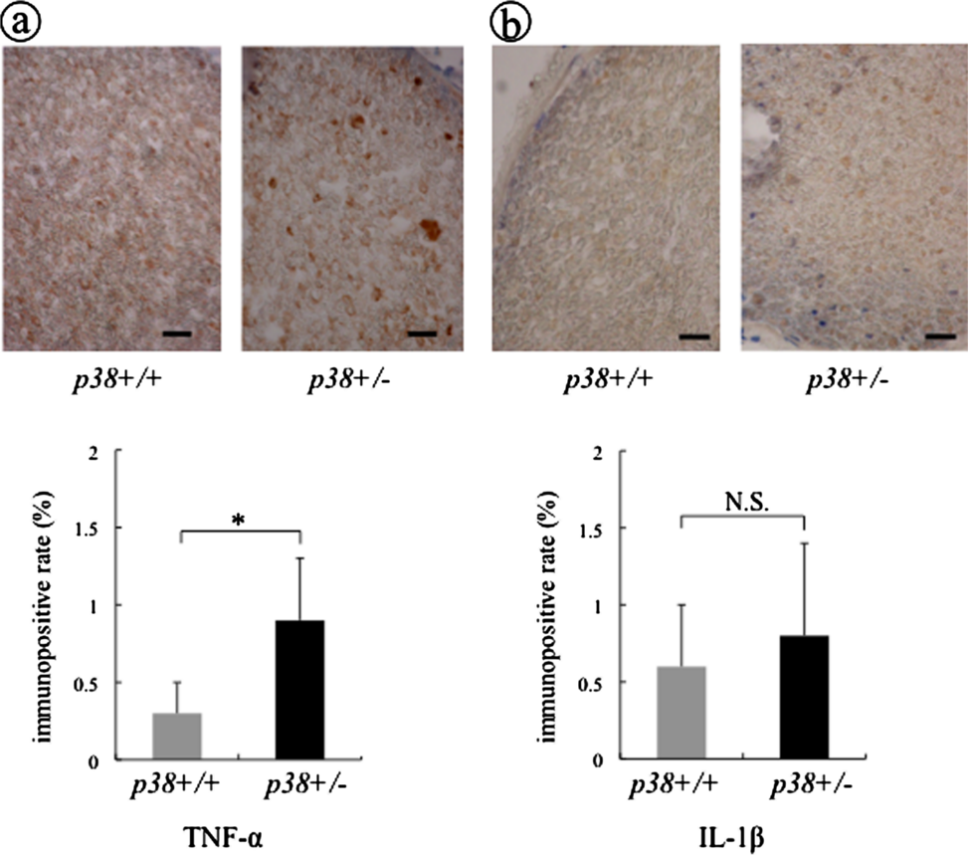
**The expression of TNF-α and IL-1β at four weeks after crush injury.** At four weeks after crush injury, specimens from both sem (p38+/−) and wt (p38+/+) mice were processed in the same way as shown in Figure 4. Scale bar indicates 50 μm. The ratio of the immunopositive area of **(a)** TNF-α and **(b)** IL-1β to the total area of the field was calculated as the immunopositive rate, respectively. Asterisks indicate significant difference between genotypes (*P <0.05) and N.S. indicates no significant difference.

**Figure 6:**
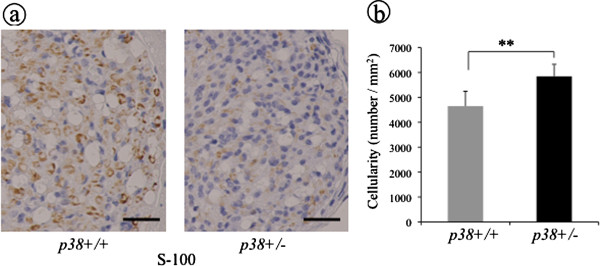
**The expression of S-100 and cellularity at four weeks after crush injury. (a)** At four weeks after crush injury, specimens from both sem (p38+/−) and wt (p38+/+) mice were processed for immunostaining using anti S-100 antibody. Scale bar indicates 50 μm. **(b)** Cellularity was evaluated by counting the number of cells per microscopic field and expressed as the cell density (N/mm^2^). Asterisks indicate significant difference between genotypes (**P <0.01).

We next investigated immunohistochemically the expression of cytokines during the late response phase (four weeks after injury). It has been shown that the expression of both TNF-α and IL-1β is downregulated at this phase. At four weeks after crush injury, TNF-α-ir lesions could be still observed in sem mice but not in wt mice. The positive rate in sem mice was significantly higher (0.9 ± 0.4%) than that of wt mice (0.3 ± 0.2%) (*P* < 0.05) (Figure 5a). IL-1β expression in sem mice (0.8 ± 0.6%) was also higher than that in wt mice (0.6 ± 0.4%), but this did not reach significance (Figure 5b). In addition, Schwann cells were stained using an anti S-100 antibody, a marker of mature Schwann cells, after which these specimens were counterstained to evaluate cellularity. S-100-ir areas were observed diffusely around axons and their intensity in wt mice was stronger than that in sem mice (Figure 6a). In addition, the cellularity in sem mice (5846.4 ± 482.5) was significantly higher than that in wt mice (4647.6 ± 598.1) (Figure 6b, *P* < 0.01). These results indicate that nerve regeneration was retarded in sem mice compared with that in wt mice.

### Western blot analysis

**Figure 7:**
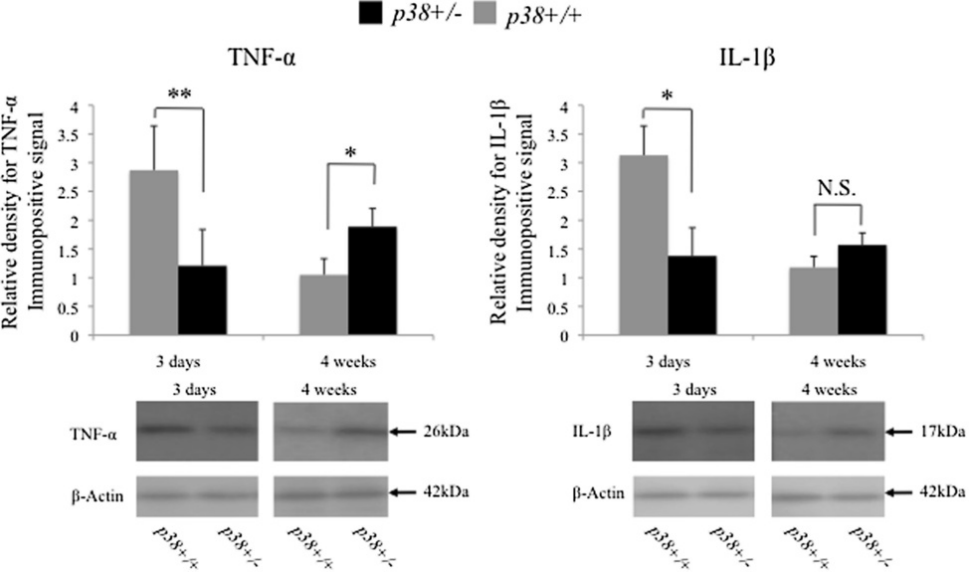
**Western blot analysis.** Protein lysates extracted from sciatic nerves from both wt (p38+/+) and sem (p38+/−) mice were examined by western blotting for TNF-α and IL-1β expression at three days and four weeks following crush injury. Expression at each time point (n = 3 samples per group) were analyzed and quantified as relative density against control.

Analysis of protein levels by western blotting revealed that, in both groups, only low levels of TNF-α and IL-1β were present in contralateral sciatic nerves (data not shown). However, the expression levels of both cytokines in wt mice was markedly increased at three days following crush injury (approximately three-fold over control values). The increase was transient and both expression levels returned to control values at four weeks after crush injury. In contrast to the changes observed in wt mice, there was no observable change in the expression of either cytokine in sem mice at three days after crush injury. However, consistent with the immunohistochemical findings, increases in the levels of TNF-α were observed at four weeks after crush (approximately two-fold over control values) in sem mice. IL-1β levels in sem mice were also slightly higher than those in wt mice. Between sem and wt mice, statistically significant differences were observed in the expression of TNF-α and IL-1β at three days after crush, and of TNF-α at four weeks after crush (*P* < 0.05, *P* < 0.01, and *P* < 0.01, respectively) (Figure 7).

## Discussion

Peripheral nerves are known to have a considerable capacity for regeneration after injury, a process regulated by several inflammatory cytokines expressed within the injured nerve. An understanding of the function of inflammatory cytokines and cytokine-regulatory proteins during nerve regeneration offers the potential for broad-reaching therapeutic intervention. Therefore, this regulated complex signaling cascade has been an area of intense investigation.

The p38 MAPK is one of the most important kinases in regulating the inflammatory signaling cascade, so that many different p38 MAPK inhibitors, all of which are competitive with ATP, have been used to investigate the physiological role of p38 MAPK *in vitro*. Of these, SB203580, the pyridinyl imidazole compound, has been widely used as a specific inhibitor of p38 MAPK. However, it was recently revealed that this inhibitor affects several unrelated kinases, therefore, incorrect conclusions might have been drawn from numerous experiments in which SB 203580 was used as a ‘specific’ p38 inhibitor [14]. Thus, it is of importance to explore ‘specific’ methods to block the phosphorylation of specific substrates mediated by p38 MAPK. This is the first report showing the physiological role of p38 MAPK *in vivo* by specifically blocking its docking-domain. Recently, we confirmed by immuno-precipitation that p38 MAPK derived from sem mice maintains interactions with transcription factors, including activating transcription factor 2 (ATF2) but not with protein kinases such as mitogen-activated protein kinase-activated protein kinase 2 (MAPKAPK2) [18]. This finding indicates that sem mice can be used to investigate limited loss-of-function of p38 MAPK. Using these mutant mice, we have confirmed that p38 MAPK insufficiency delays nerve regeneration following crush injury, both histologically and functionally.

George *et al*. [29] reported that TNF-α was increased in the injured mouse sciatic nerve on day 1 and returned to the control level on day 7 after crush injury. There are also several reports showing that endoneurial TNF protein expression peaked early and transiently in mouse sciatic nerves at the site of lesion [30,31]. It has been reported that TNF-α functioned as a primary mediator to initiate the cascade of nerve degenerative events, inducing many changes in Schwann cells and neuron gene expression [2,3]. Consequently, TNF-α is recognized as a biomarker of Wallerian degeneration in the injured nerve. Direct injection of TNF-α into uninjured nerves causes demyelination, macrophage infiltration, and pain, mimicking changes observed in injury [3,32].

On the other hand, IL-1β is known to have several beneficial effects on the nervous system, promoting Schwann cell proliferation [33], neuron survival [34,35], synthesis of nerve growth factor (NGF) [36], and oligodendrocyte remyelination [37]. Furthermore, it has also been reported that IL-1β overcomes neurite outgrowth inhibition by deactivating the small GTPase RhoA, which results in promoting nerve regeneration. Temporin *et al*. [12] reported that expression of IL-1β was increased one day after nerve injury, and that this increase in expression persisted for 7 days. Taken together, it is considered that the expression of both TNF-α and IL-1β is indispensable for the early phase of nerve regeneration after injury.

In this study, the expression of TNF-α and IL-1β in p38 MAPK-insufficient sem mice was significantly lower than that in wt mice at three days after crush injury. This result implies that p38 MAPK functions as an upstream signal of TNF-α and IL-1β *in vivo*, and also suggests that reduced expression of these cytokines may be an obstacle to fulfill Wallerian degeneration and subsequent regeneration in the early phase after injury. It has been reported that adult dorsal root ganglion neurons in culture do not extend neurites in cryosections of normal sciatic nerve, but do on cryosections of pre-degenerated nerves [38]. Wallerian degeneration, which is induced by TNF-α, is required for nerve regeneration. Therefore, this might be one of the mechanisms by which regeneration is retarded in the mutant mice. However, intriguingly, we observed elevated TNF-α expression at four weeks after crush injury in sem mice. It is well known that the activation of p38 MAPK occurs transiently followed by its dephosphorylation. However, in sem mice, the expression of phosphorylated p38 MAPK is prolonged due to an inability to bind its substrates, such as MAPKAPK2. This impairment of p38 MAPK autorepression might be one of the reasons for the elevation of TNF-α at four weeks after crush injury. We consider that this prolonged expression of TNF-α might be deleterious for successful nerve regeneration. Agents that target TNF-α and IL-1β have received attention as potential therapeutics for nerve injury, due to their inflammatory effect. However, whether clinical application of neutralizing antibody and/or receptor antagonist of these would be effective has not reached consensus. It is likely that TNF-α and IL-1β play different roles at different phases of nerve regeneration. Therefore, further studies are required to elucidate the molecular mechanisms regulating inflammation in the nervous system.

Previously, it has been reported that the expression of Caspase-3 is upregulated in the endoneurium at ten days after nerve transection [39], and that Tenascin-C demonstrates peak expression during the first two weeks after nerve injury and becomes downregulated subsequently when axons regenerate [40,41]. Our results in wt mice are consistent with these previous data, and indicate that elevation of Caspase-3 followed by expression of Tenascin-C are important early responses in injured peripheral nerves. In this study, expression both Caspase-3 and Tenascin-C was significantly lower in sem mice than that in wt mice. This result supports the idea that the inhibition of p38 MAPK retarded Wallerian degeneration and subsequent axonal regeneration after nerve injury.

We also confirmed that the expression of S-100 was inhibited in sem mice and the cellularity in sem mice was higher than that in wt mice at four weeks after crush injury. This decreased expression of S-100 in the late phase after injury was consistent with previous findings [42] and the increase in cellularity is not surprising, given the attendant changes in the progressing degenerating-regenerating nerve [43]. We consider these changes to also be secondary to the insufficiency of p38 MAPK and of TNF-α and IL-1β in sem mice.

In this report, we describe the specific inhibition of p38 MAPK *in vivo* without the use of chemical inhibitors, and we have confirmed for the first time that the inhibition of p38 MAPK delays nerve regeneration following crush injury, both histologically and functionally. Our findings suggest that molecular targets to regulate the repair of damaged peripheral nerves might exist in the p38 MAPK signaling pathway. We did not evaluate either the intrinsic responses of the neurons in dorsal root ganglia or changes of non-myelinated axons in sciatic nerves in this study. However, our current findings support the approach of specific modulation of inflammatory responses in the treatment of peripheral nerve injury. Further studies are now required to clarify the physiological role of the p38 MAPK signaling pathway for nerve regeneration.

## Conclusions

This is the first study of the physiological role of p38 MAPK in nerve regeneration that does not rely on the use of pharmacological inhibitors. Our results indicate that p38α insufficiency may cause an inflammatory process disorder, resulting in a delay of histological and functional nerve recovery following crush injury. We conclude that p38 MAPK has an important physiological role in nerve regeneration and may be important for controlling both initiation of inflammation and recovery from nerve injury.

## Abbreviations

EPL: Experimental print length; ETS: Experimental toe spread; MAPK: Mitogen-activated protein kinase; MAPKAPK2: Mitogen-activated protein kinase-activated protein kinase 2; MKK: MAPK kinase; MKKK: MAPK kinase kinase; NGF: Nerve growth factor; NPL: Control print length; NTS: Control toe spread; OCT: Optimal cutting temperature; PL: Print length; RICK: Rip-like interacting caspase-like apoptosis-regulatory protein kinase; RIP: Receptor interacting protein kinase; sem mice: *sevenmaker* type Mapk14 mice; SFI: Sciatic functional index; TS: Toe spread; wt: wild type.

## Competing interests

The authors declare that they have no competing interests.

## Authors’ contributions

All authors read and approved the final version of the manuscript.
